# Prediction of aesthetic reconstruction effects in edentulous patients

**DOI:** 10.1038/s41598-017-17065-y

**Published:** 2017-12-22

**Authors:** Fusong Yuan, Cheng Cheng, Ning Dai, Yuchun Sun

**Affiliations:** 1 Center of Digital Dentistry, Peking University School and Hospital of Stomatology; Department of Prosthodontics, Peking University School and Hospital of Stomatology; National Engineering Laboratory for Digital and Material Technology of Stomatology; Research Center of Engineering and Technology for Digital Dentistry of Ministry of Health; Beijing Key Laboratory of Digital Stomatology, Beijing, China; 2 College of Mechanical and Electrical Engineering, Nanjing University of Aeronautics and Astronautics, Nanjing, China

## Abstract

The aim of the study is to establish a virtual prediction method to predict aesthetic reconstruction effects in edentulous patients. The facial soft tissue surface data before and after wearing complete dentures of ten edentulous patients were acquired with a facial Three-dimension scanner. Then, the two sets of scanned data were entered into the same coordinate system. Manual interaction was performed to extract the external boundary of the perioral appearance deformation area, and the proportional relationships of key facial anatomical features were measured. A virtual prediction software module was developed based on back-propagation neural networks and a Laplacian deformation algorithm. Virtual prediction of the aesthetic reconstruction effects in the overall appearance of the lower third of the face was performed in 10 edentulous patients. The mean accuracy of the virtual predictions was approximately 0.769 ± 0.205 mm, and there were statistically significant differences between the 10 patients (p < 0.05). The scope of the changes in facial appearance of edentulous patients was smaller than the scope of the lower third of the face. This method can achieve the virtual prediction of soft tissue appearance in the lower third of the face after wearing complete dentures to an extent.

## Introduction

When humans lose all their teeth, the condition is known as missing dentition or complete edentulism. Edentulous patients experience a loss of basic masticatory function, and the progressive absorption of alveolar bone. Different degrees of collapse occur in facial soft tissue, with the lack of teeth and sufficient support from alveolar bone. This leads to shortening in the lower third of the face, deeper wrinkles, drooping of the labial commissures, and other changes^1^. The aesthetic reconstruction of facial appearance in edentulous patients is one of the crucial objectives in complete denture prosthodontics.

The wearing of complete dentures by edentulous patients is equivalent to local plastic surgery. To achieve satisfactory aesthetic restoration, dentists need to be able to predict the patient’s post-restoration facial appearance and changes in facial soft tissue prior to performing restoration. This prediction can then be incorporated into the design of the artificial dentition and denture base for complete dentures. Currently, the prediction of facial deformation is mainly dependent on the dentist’s subjective judgment and experience, rather than quantitative scientific analysis. A number of researchers have striven to achieve the quantitative prediction of changes in facial soft tissue after maxillofacial restoration^2^. These studies can be divided into three types based on their calculation methods, the mass-spring model (MSM), the finite element model (FEM), and the mass tensor model (MTM).

Due to the physical implications and computational efficiency of MSM, it was the earliest method to be applied to real-time facial simulation. Koch *et al*. used computed tomography (CT) data to reconstruct facial and skull models, and established a MSM between the facial and skull models, in order to perform deformation simulations^3^. Due to the complex structural mechanics of facial soft tissue, MSM often leads to the occurrence of hyperelasticity during the simulation process, which can lead to greater distortion.

While the FEM has relatively good biomechanical relevance, its computational costs are high. Sarti *et al*. proposed a computationally-intensive FEM based on voxel elements^4^. Zachow *et al*. proposed the simulation of soft tissue models using FEM based on tetrahedral elements to enhance computation speed, and applied this approach for clinical use^5^. Gladilin applied FEM based on tetrahedral elements on facial muscles^6^. Due to the high computational load and long computing time of FEM, many studies have focused on optimizing the computational strategies and models^7,8^.

MTM was initially developed to balance computational speed and accuracy. Mollemans *et al*. applied MTM to the simulation of facial soft tissue deformations for maxillofacial surgery^9^. They used CT data to reconstruct facial and skull models, and applied MTM to simulate post-surgical skull displacement and facial deformations. MTM can be regarded as a hybrid of FEM and MSM. While the model retains the simple structure of MSM, it also has the biomechanical relevance of FEM. Nevertheless, when analyzing soft tissue models, CT scans of the soft tissue are needed to determine the boundary conditions of the model. However, the radiation dose associated with CT scans is potentially harmful to the human body.

Artificial neural networks have been widely applied in various fields, and are particularly suitable for solving multi-parameter operations and non-linear interpolation problems. Back-propagation (BP) neural networks are typical artificial neural networks that can solve complex non-linear problems. Scott *et al*. employed artificial neural networks to predict the properties of ceramic materials^10^. Neural networks have also been used to accurately predict the performance of highly complex materials^11,12^.

Our research group has previously established a deformation algorithm based on the as-rigid-as-possible (ARAP) technique for the simulation of edentulous maxillofacial soft tissue after wearing complete dentures. The approach has high computational accuracy and efficiency, and does not require CT scanning^13^. The present study combined this approach with facial color 3D scanning technology to establish a data registration method suitable for quantitative investigation of changes in facial soft tissue appearance before and after the wearing of complete dentures in edentulous patients. The method was then used to measure the boundaries of the pre-operative and post-operative appearance deformation areas in 10 Chinese edentulous patients, followed by the preliminary construction of statistical models for these boundaries. Based on the resulting data, a virtual prediction algorithm was established using BP neural networks and a Laplacian deformation algorithm to determine the overall aesthetic reconstruction effects in the lower third of maxillofacial soft tissues. Virtual prediction was performed on the overall post-operative 3D appearance of the lower third of the facial soft tissue in 10 Chinese edentulous patients, and the predictions were evaluated by quantitative comparison with the actual post-operative appearance. Our results have laid the foundation for studies on the accurate digital reconstruction of oral and maxillofacial aesthetics.

## Materials and Methods

### Experimental equipment and software

The current study used a facial 3D scanner (Facescan, 3D Systems, USA) with a scanning precision of 0.1 mm. Geomagic Qualify 2012 (Geomagic Inc., USA) 3D analysis software was used, as were an Intel^®^ Core^TM^ i5–3550 processor with 8GB RAM, a 1TB hard-drive, and a ViewSonic VG920 color monitor. The MicrosoftVisual C^++^ 6.0 (Bell Labs, USA) programming language was utilized.

### Experimental methods

#### Sample collection

Ten patients with edentulous jaws [five males (50.0%) and five females (50.0%)] with a mean age of 73.2 ± 4.3 years (range 68–80 years) were enrolled in the study. They underwent complete denture prosthodontics at the Department of Prosthodontics of the Peking University School of Stomatology, Beijing, China. Inclusion criteria for patients were edentulous patients who wore complete dentures made in the Department of Prosthodontics of the Peking University School of Stomatology. Patients were excluded if they were rated ≤20 points on the Revised Hasegawa Dementia Scale, which is a scale of dementia, or if they had symptoms of severe myopia or daltonism. No patient was excluded after these preliminary examinations. From preliminary tests, the sample size (n = 10) was determined with a power analysis to provide statistical significance (a = 0.05) at 80% power. The study was approved by the Bioethics Committee of the Stomatological Hospital of Peking University ((No.PKUSSIRB-201627042. Date: 27/6/2016). All of the experimental protocols and procedures were approved by the licensing committee and performed in accordance with the approved guidelines and regulations. The patients were informed that the extracted teeth would be used in the *in vitro* study, and informed consent was obtained from all of the subjects.

#### Data acquisition

**Figure 1:**
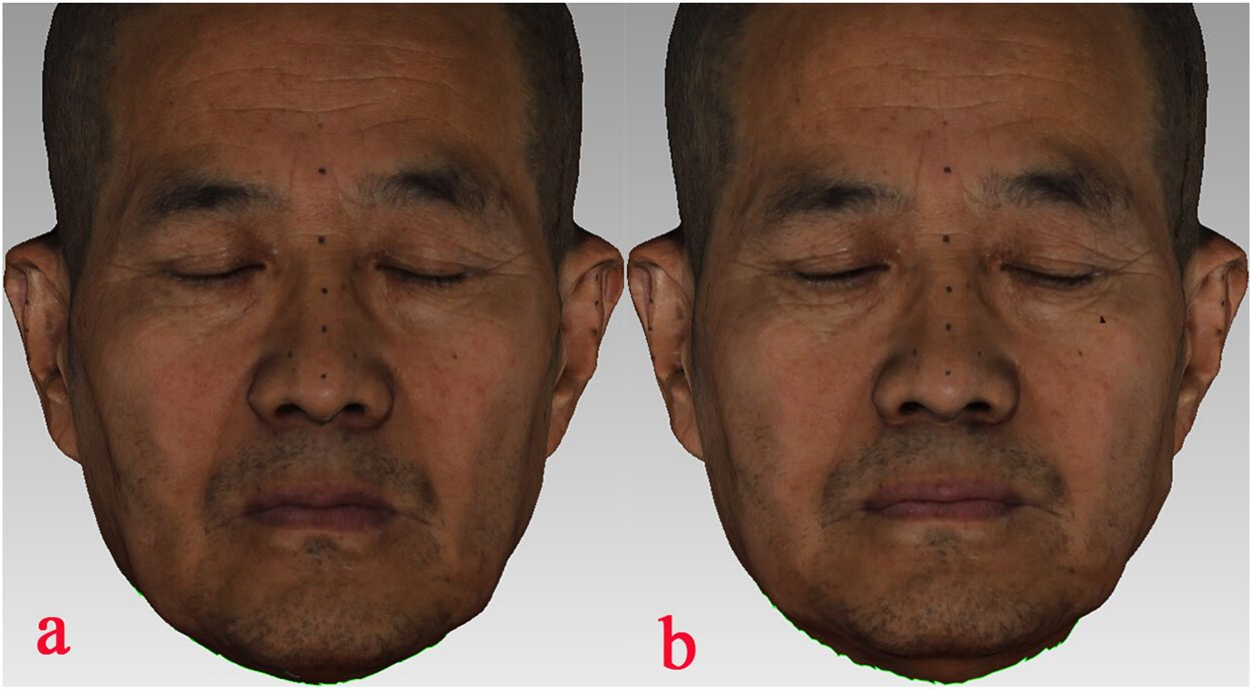
Facial 3D color scanning data of an edentulous patient before denture insertion: (**a**) before inserting the denture; (**b**) after inserting the denture.

The patients were asked to sit upright with their heads positioned naturally and their mandibles in the mandibular postural position. A black marker pen was used to make three circular marks on a relatively flat area on the bilateral tragus and the nasal tip. A facial 3D color scanner was used to acquire true color 3D data from the patients’ faces before and after wearing complete dentures. The pre-operative data were labeled A, and the post-operative data were labeled B. All data were stored in OBJ format (Fig. 1).

#### Data registration

**Figure 2:**
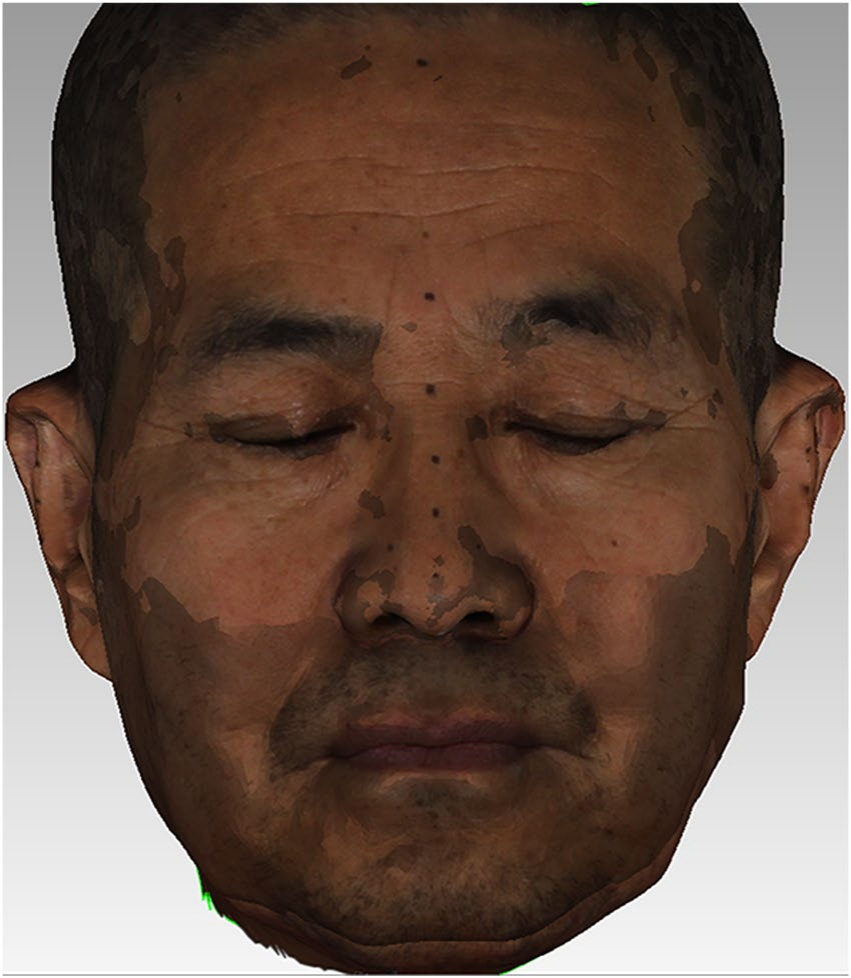
Registration results of facial 3D scan data of edentulous patients before and after denture insertion.

A and B data were imported into the Geomagic 2012 software. The approximate center of the three black marks on the bilateral tragus and nasal tip were selected via manual interaction. Alignment between A and B data was performed using the “n-point registration mode” in “manual registration”, with A as the reference. Using the upper third of the face as the shared area, the “best fit” registration command was then applied to register the two sets of scan data on the same coordinate system (Fig. 2).

**Figure 3:**
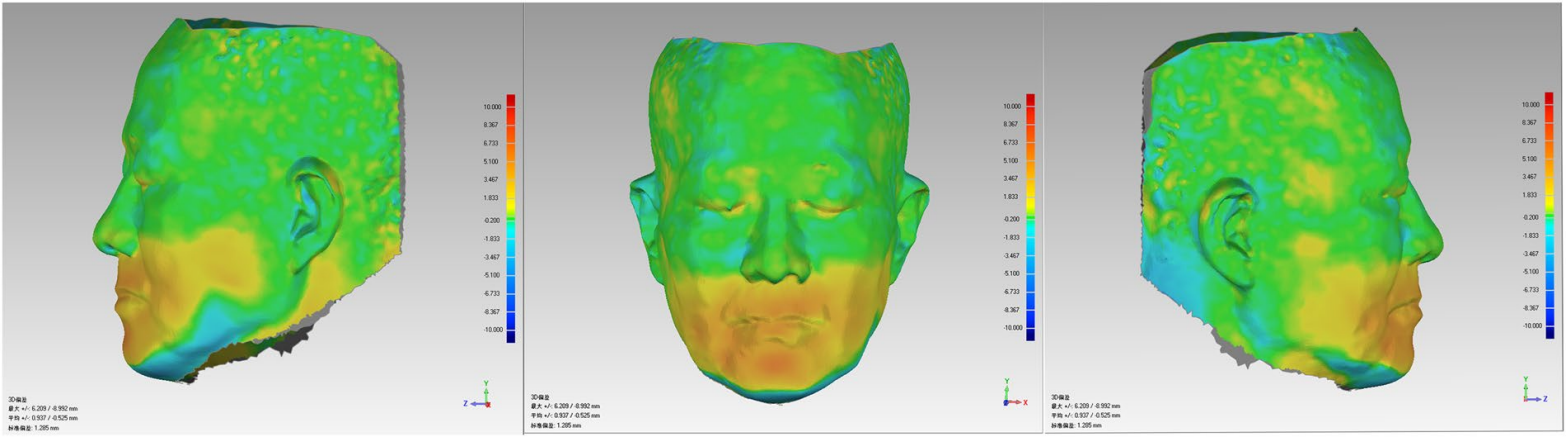
The 3D deviation analysis of facial 3D scan data of edentulous patients before and after denture insertion.

3D deviation analysis. The “3D deviation analysis” command in the Geomagic 2012 software was applied. Based on the visual acuity of the human eye, the critical value was set as ± 0.2 mm^2^, and A data were used as the reference to perform deviation analysis on B data (Fig. 3).

Statistical model for discerning post-operative deformation area. The following measurements were taken from the 10 patients: Perpendicular distance between the uppermost boundary of the appearance deformation area and the ala-tragus plane (plane formed by joining the centers of the bilateral tragus and the outermost points on the nasal ala); perpendicular distance of the bottommost boundary and soft tissue menton (the lowest point on the chin); ratio between the lengths of the maximum left/right boundary–ipsilateral commissure line, and the ipsilateral tragal center–commissure line. Each distance above was measured 10 times by one operator and the average value and standard deviation was calculated. This method was applied in all 10 patients. The measurement data was imported into SPSS 13.0, and analysis was performed via one-way analysis of variance (ANOVA).

#### Virtual prediction

Virtual prediction software was developed, and virtual prediction of the overall appearance in the lower third of the facial soft tissue in 10 edentulous patients after wearing complete dentures was performed. To enhance data operability and computational efficiency, and to simplify the point cloud models, it was necessary to construct standardized feature templates to replace the 3D scan data of the facial deformation area. Prior to constructing the feature templates, the upper third of the face was specified as the shared area, and the iterative closest point (ICP) method was employed to register the edentulous (pre-operative) and dentate (post-operative) models on the same coordinate system.

**Figure 4:**
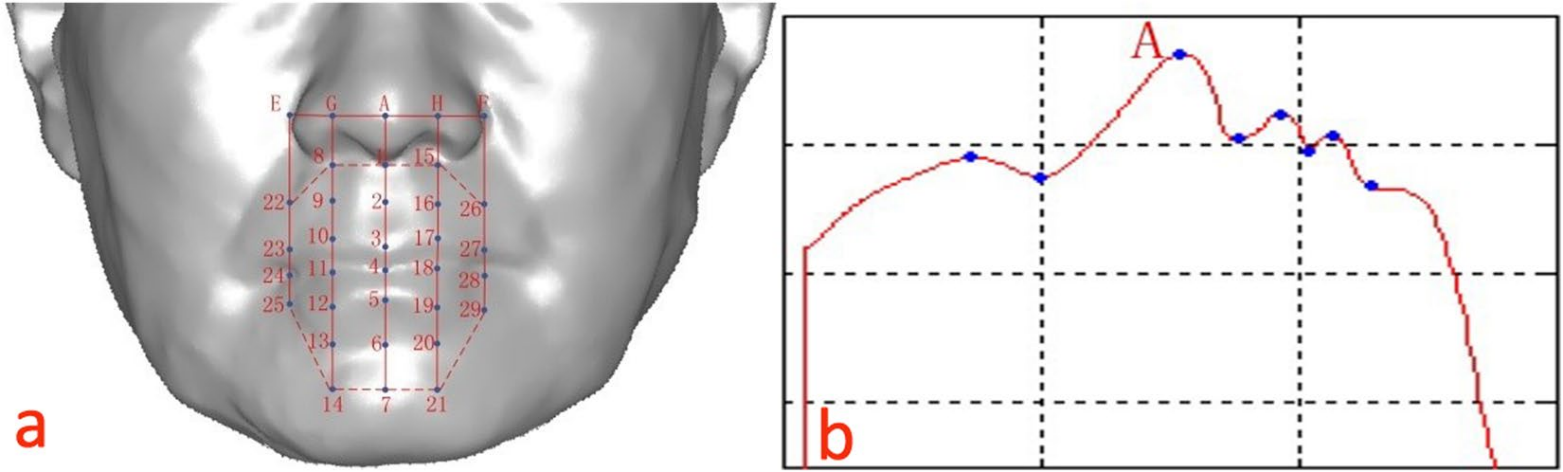
Construction of the feature template. (**a**) Isoline passing through the nasal tip. (**b**) Feature template.

Based on the corrected facial model, we constructed the feature template shown below in Fig. 4(a). The main sequence of events included the following steps:

1. In the coordinate system shown in Fig. 4, the maximum vertex A was identified in the Z-axis.
2. The vertical isoline that passed through point A in the Y-axis was obtained, as shown in Fig. 4(b). Based on the curvature changes, the feature points under the nose and on the lips were located and labeled 1, 3, 4, and 5. The change in curvature was based on the mean curvature and can be given as follows:
  1 $$H=\frac{1}{n}\sum _{i=1}^{n}{K}_{i}\,i=1,2,\,\ldots ,\,n\,$$
  2 $${K}_{i}=\frac{2\cdot ({p}_{j}-{p}_{i})\cdot \mathop{n}\limits^{\rightharpoonup }}{({p}_{j}-{p}_{i})\cdot ({p}_{j}-{p}_{i})}\,$$
  where *H* is the mean curvature, *n* is the number of points in the neighborhood, ${K}_{i}$ is the curvature of the surface passing through the center ${p}_{i}$ and the points ${p}_{j}$ in the neighborhood, and $\mathop{n}\limits^{ \rightharpoonup }$ is the normal vector of ${p}_{i}$. The midpoint between points 1 and 3, and the symmetric points 6 and 7 relative to points 1 and 2 were computed on the XY plane.
3. The horizontal isoline in the X-axis that passed through point A was taken. Then, the feature points E and F on both sides of the nose were located based on the change in curvature. The midpoint G between A and E, and the midpoint H between A and F were computed on the XY plane.
4. Similar to Step (2) above, the vertical isoline that passed through points E, G, H, and F was obtained, and the feature points were located based on the change in curvature. Lastly, the feature template composed of points 1–29 was obtained.

Construction of elastic prediction model for soft tissue. Principal component analysis (PCA) is an important statistical method that involves the transformation of multi-index problems into a smaller number of comprehensive indexes. It can convert high-dimensional problems into low-dimensional problems for further processing, making the problems simpler. Furthermore, these comprehensive indexes are mutually independent, and contain the vast majority of the information contained within the original indexes.

The basic idea of PCA can be summarized as follows: An orthogonal transformation can be used to convert the original random variables with correlated components into new variables with uncorrelated components. From an algebraic perspective, it implies converting the covariance matrix of the original variables into a diagonal matrix. From a geometric perspective, it involves transforming the original variable system into a new orthogonal system, such that it points towards the orthogonal direction of where the sample points are the most spread out, thus achieving dimensionality reduction on a multi-dimensional variable system. From the perspective of feature extraction, PCA is equivalent to an extraction method based on the minimum mean square error. The completed PCA model is also the facial elastic deformation prediction model, which can predict the elastic deformation of the edentulous model feature template.

Deformation simulation based on Laplacian deformation technique. Laplacian mesh processing uniformly regards its processing target as a discrete scalar field defined on the vertex of the 3D mesh model. It uses the differential properties of this scalar field on a discrete face to effectively express the local features of this field. After performing certain operations, it reconstructs the differential properties using a global optimization process into a scalar field containing the initial meaning.

For each vertex ${{\rm{v}}}_{{\rm{i}}}$ in the mesh model, we can use the linear combination of its adjacent edge vectors to define the differential coordinates ${{\rm{d}}}_{{\rm{i}}}$ of one vertex via the following equation:

3 $${{\rm{d}}}_{{\rm{i}}}={({{\rm{d}}}_{{\rm{i}}}^{({\rm{x}})},{{\rm{d}}}_{{\rm{i}}}^{({\rm{y}})},{{\rm{d}}}_{{\rm{i}}}^{({\rm{z}})})}^{{\rm{T}}}={\rm{L}}({{\rm{v}}}_{{\rm{i}}})={\sum }_{{\rm{j}}\in {\rm{N}}({\rm{i}})}{{\rm{w}}}_{{\rm{ij}}}({{\rm{v}}}_{{\rm{i}}}-{{\rm{v}}}_{{\rm{j}}})$$

where ${{\rm{d}}}_{{\rm{i}}}$ is also known as the Laplacian coordinates, and ${{\rm{w}}}_{{\rm{ij}}}$ is the weight of the edge (ij). The Laplacian coordinates (${{\rm{d}}}_{{\rm{i}}}$) of all mesh vertices can be combined to give matrix ${\rm{D}}={({{\rm{d}}}_{1}^{{\rm{T}}},{{\rm{d}}}_{2}^{{\rm{T}}},\ldots ,{{\rm{d}}}_{{\rm{n}}}^{{\rm{T}}})}^{{\rm{T}}}=({{\rm{d}}}^{({\rm{x}})},\,{{\rm{d}}}^{({\rm{y}})},\,{{\rm{d}}}^{({\rm{z}})})$, and we can use the Laplacian matrix ${\rm{L}}=({{\rm{l}}}_{{\rm{ij}}})$ to more conveniently express Equation (4):

4 $${\rm{D}}={\rm{LV}},{\mathrm{where}l}_{{\rm{ij}}}=\{\begin{array}{c}{\sum }_{{\rm{j}}\in {\rm{N}}({\rm{i}})}{{\rm{w}}}_{{\rm{ij}}},i=j,\\ -{{\rm{w}}}_{{\rm{ij}}},\,{\rm{j}}\in {\rm{N}}({\rm{i}}),\\ 0,\,{\rm{otherwise}}.\end{array}$$

The Laplacian matrix L is also known as the Laplacian operator defined on mesh M.

By using ${{\rm{v}}}_{{\rm{i}}}$ to denote the *i* ^th^ vertex in the mesh model, and ${{\rm{\delta }}}_{{\rm{i}}}$ to denote the Laplacian coordinates corresponding to ${{\rm{v}}}_{{\rm{i}}}$, we can compute the center of the neighborhood points for this vertex as follows:

5 $${\bar{{\rm{v}}}}_{{\rm{i}}}=\frac{1}{{{\rm{d}}}_{{\rm{i}}}}\sum _{{\rm{j}}\in {\rm{N}}({\rm{i}})}{{\rm{v}}}_{{\rm{j}}}$$

where ${\bar{{\rm{v}}}}_{{\rm{i}}}$ represents the center of all vertices directly adjacent to the vertex ${{\rm{v}}}_{{\rm{i}}}$, and ${{\rm{d}}}_{{\rm{i}}}$ represents the number of vertices directly linked to the *i* ^th^ point. The Laplacian operator represents the amount of displacement of the current point from the center of all its neighboring points.

Under uniform weighting, the Cartesian coordinates of the triangular mesh can be transformed into Laplacian coordinates using the following equation:

6 $${{\rm{\delta }}}_{{\rm{i}}}={\rm{L}}({{\rm{v}}}_{{\rm{i}}})={{\rm{v}}}_{{\rm{i}}}-\frac{1}{{{\rm{d}}}_{{\rm{i}}}}{\sum }_{{\rm{j}}\in {\rm{N}}({\rm{i}})}{{\rm{v}}}_{{\rm{j}}}$$

Let A be the adjacency matrix of the mesh. Then, according to discrete mathematics, A can be expressed as follows:

7 $${A}_{{\rm{ij}}}\{\begin{array}{c}{\rm{1}},\,(i,\,{\rm{j}})\in {\rm{edge}}\\ {\rm{0}},\,\,{\rm{otherwise}}\end{array}$$

Let D be the diagonal matrix, where ${{\rm{D}}}_{{\rm{ii}}}={{\rm{d}}}_{{\rm{i}}}$. Thus, the matrix for the transformation of Cartesian coordinates to Laplacian coordinates, *i*.*e*., matrix L, can be expressed as L=I − D^−1^ A. Lastly, the new coordinates can be given by:

8 $$[\begin{array}{c}{\rm{L}}\\ {\rm{I}}\end{array}]{\rm{V}}^{\prime} =[\begin{array}{c}{\rm{\delta }}\\ {\rm{w}}\end{array}]$$

where I is the weights of the control points, w is the coordinates of the control points, and ${\rm{V}}^{\prime}$ is the coordinates of the final model points. Based on the constraints, the feature points of the feature template and the points in the deformation were used to construct the Laplacian equation. Then, deformation simulation was performed on the edentulous facial mode according to changes in the feature points.

Comparative evaluation of virtual prediction results. 10 virtual predictions were performed for each patient by one operator. The virtual prediction data and the patients’ post-operative scan data were imported simultaneously into the Geomagic 2012 software. By specifying the upper third of the face as the shared area, the “best fit” registration command was used to register both datasets onto the same coordinate system, and deviation analysis was performed on the appearance deformation area. After that, each patient got 10 sets of error values, and the average and standard deviation were calculated. Each patient was treated with this method, and then all the error values of 10 patients were statistically analyzed.

## Results

With regard to the appearance deformation area of the 10 patients before and after wearing complete dentures, the maximum value of the distance between the uppermost boundary and the ala-tragus plane was 2.402 mm, the minimum value was 0.181 mm, and the mean value was 1.221 ± 0.657 mm. The maximum value of the distance between the bottommost boundary and soft tissue menton was 1.518 mm, the minimum value was 0.029 mm, and the mean value was 0.735 ± 0.491 mm. The distance from the left maximum boundary to the commissure = a straight line from the left tragal center to the commissure *(0.794 ± 0.021). The distance from the right maximum boundary to the commissure = a straight line from the right tragal center to the commissure *(0.789 ± 0.019). One-way ANOVA of the data showed that the differences were not statistically significant (*P* > 0.05).

3D deviation analysis indicated that when the virtual predictions of the 10 patients were compared to the post-operative model, the maximum value of the error was 1.090, the minimum value was 0.480, and the mean value was 0.769 ± 0.205. Independent-samples *t*-test results showed that there were statistically significant differences in the error of the virtual prediction results for facial soft tissue among the 10 patients (Table 1).

**Table 1:** Statistical analysis of virtual prediction accuracy (independent-samples *t*-test).

	Levene’s test for equality of variance		*t*-test for equality of means						
	F	*P*	t	df	*P*(2-tailed)	Mean difference	Std. error difference	95% confidence interval of the difference	
								Lower	Upper
Equal variances assumed	18.973	< 0.001	8.785	18	<0.001	0.569	0.065	0.433	0.705
Equal variances not assumed			8.785	9	<0.001	0.569	0.065	0.422	0.715

df, degrees of freedom.

## Discussion

Studies have shown that there are proportional relationships in the human face. Of these, vertical proportional relationships can be determined using the trisection or the bisection method. The trisection method implies that the upper third (trichion to the glabella), middle third (glabella to the subnasale), and lower third (subnasale to the soft tissue menton) should be of approximately equal distances. The bisection method implies that the distance between the subnasale and the menton should be approximately equal to that between the outer canthus and the commissure. The soft tissue appearance of the lower third of the face has important reference value when designing the fullness of the anterior dental region for complete dentures. However, there are currently no studies on the prediction of external facial appearance after complete denture prosthodontics. The current study is the first to combine BP neural networks with a Laplacian deformation technique to devise a novel method for the prediction of facial appearance after complete denture prosthodontics.

During the experimental process, the prediction results of the BP neural network were observed by adjusting the number of hidden layer nodes, and the errors of the prediction results were compared to determine the number of hidden layer nodes in the final neural network. The final number of hidden layer nodes selected in this study was 20, and the mean error rate was 26.06%. The individual run times of the three programs (feature template construction, elastic deformation prediction, and deformation simulation) were all less than 3 s, and the total run time was less than 10 s. Chabanas *et al*. employed FEM to simulate facial soft tissue deformation after maxillofacial treatment, and the computational time was 2–7 min^14^. The deviation between the deformation results and the post-treatment model was within 2 mm, and their accuracy levels were similar to those observed in the current study. However, the computational time was more efficient in the current study.

Currently, FEM and MTM are the most frequently used models for the simulation of facial soft tissue. In these methods, patients need to undergo CT scans to determine the boundary conditions of the model, including the soft tissue and bone boundaries. However, the radiation dose associated with CT scans is potentially harmful to the human body. The current study did not require a skull model, but instead relied on facial model data for prediction and simulation. The method can facilitate the design of treatment plans, as it is able to provide a pre-operative prediction of changes in a patient’s post-operative appearance and facial soft tissue. In addition, it provides a 3D model to the dentists and patients, which enables them to understand the changes after complete denture prosthodontics more intuitively. Furthermore, dentists can formulate individualized and appropriate dental arrangements for complete dentures according to the facial deformation.

Although soft tissue changes in the lower third of the face have crucial implications in the design and creation of complete dentures, researchers have yet to quantitatively describe the boundaries of soft tissue changes in the lower third of the face. This has led to difficulties in relevant quantitative research within this field. The present study employed true color 3D scanning and 3D deviation analysis to preliminarily establish a statistical model for the 3D boundaries of the appearance deformation area in the lower third of the face after wearing complete dentures. This statistical model can objectively and quantitatively reflect the visually discernible changes in the boundaries of the lower third of the face before and after wearing complete dentures. This method will lay the foundation for future in-depth studies on facial changes in edentulous patients.

The results of the current study indicate that changes do not occur in the entire lower third of the face after complete dentures are worn. Nevertheless, due to an insufficient sample size and other factors, the study had certain limitations. Increasing the sample size will enable us to obtain more representative values to use as population statistics. Other factors related to error are detailed below.

### Data acquisition error

The Facescan facial scanner used in this study is a structured light scanner. This scanner uses white structured light scanning technology, and one of its technical features is that sources of white light have high biosafety. Furthermore, the two-sided mirror imaging device enables rapid and sequential acquisition of a series of phase-shifting fringe images from different angles in one projection. Hence, it has a relatively high scanning efficiency of approximately 0.3 s, and scanning precision of approximately 0.1 mm^15^. Due to these scanning characteristics, the surface features of the scanned faces, including color, brightness, and roughness, and the stability of the subject during scanning may affect scanning accuracy. Although data registration and stitching algorithms can be utilized by the scanning software to compensate for the effects of these factors on the coordinate changes of the data-points, reductions in the final accuracy are still unavoidable^16^. However, the minimum resolution discernible by the naked eye is 0.2 mm, hence data acquired using this facial scanner can be used to evaluate changes in appearance. In addition, breathing, heart beats, and other factors can also lead to micro-movements in the face. A study has shown that when the sampling frequency was >60 Hz, the effects of micro-movements of the human body on scanning accuracy could be maintained within 30 µm^17^. Micro-movements may still have an impact on the scanning accuracy of facial scanners. In subsequent studies, scanners with higher scanning speed should be used to improve the accuracy of data acquisition.

### Registration measurement errors during experimental software operation

In order to achieve the registration and measurement of different datasets, the present study had to utilize the processing software that came with the scanner, and reverse engineering software such as Imageware and Geomagic. The registration algorithms used in the above software are all ICP algorithms. The ICP algorithm was proposed by Besl and Mckay in 1992^18^. It is the foundation of all point cloud registration algorithms, and can effectively resolve the problem of matching free-form curves. The original ICP algorithm was groundbreaking in point cloud matching. However, the actual establishment of the best matching point is extremely time-consuming. Moreover, the ICP algorithm often has high requirements for the initial positions of the point cloud views to be registered—otherwise the algorithm would be prone to obtaining local optima, which would lead to registration errors^19^. In addition, the experimenter’s familiarity with software operation, understanding of different functions, and daily mental state may affect the accuracy of registration and measurement, thereby influencing the accuracy of the experimental results.

### Limitations of the statistical model

The present study used visual inspection and manual interaction to extract the boundaries of the appearance deformation area in the lower third of the face in 10 clinical edentulous patients. Based on these data, a preliminary mathematical model was established for the 3D boundary of the appearance deformation area in the lower third of the face after edentulous patients wore complete dentures. As this was an exploratory study, the number of cases was small, which would also affect the accuracy of the mathematical model to a certain extent. A larger sample size would ensure higher accuracy of the statistical model. Moreover, associations should be made with the elasticity coefficient of the local soft tissue for the classification of the relevant indicators, in order to further strengthen the accuracy of the statistical model.

### Future research plans

Based on the limitations of the current method, appropriate measures will be taken to address each shortcoming in future research. The number of cases included in a subsequent study will be increased. Grouped analysis will also be performed based on the elasticity features of the lower third of the face. Furthermore, additional specialized scanning and software training will be undertaken by the experimenters, in an effort to reduce the effects of erroneous operations and experimenters’ emotional changes on the measurement results.

## Conclusions

The extent of changes in appearance caused by insertion of complete dentures in edentulous patients was roughly below the base of the nose and above the menton (This range was less than 1/3 of the area below the surface), and the anterior four-fifth of the bilateral lines connecting the midpoint of each tragus with the ipsilateral angle of the mouth. And this method can achieve the virtual prediction of soft tissue appearance in the lower third of the face after wearing complete dentures to an extent.
